# Ursolic acid: a natural modulator of signaling networks in different cancers

**DOI:** 10.1186/s12935-022-02804-7

**Published:** 2022-12-10

**Authors:** Sameen Zafar, Khushbukhat Khan, Amna Hafeez, Muhammad Irfan, Muhammad Armaghan, Anees ur Rahman, Eda Sönmez Gürer, Javad Sharifi-Rad, Monica Butnariu, Iulia-Cristina Bagiu, Radu Vasile Bagiu

**Affiliations:** 1 Department of Healthcare Biotechnology, Atta-Ur-Rahman School of Applied Biosciences, National University of Sciences and Technology, Islamabad, Punjab Pakistan; 2 Faculty of Pharmacy, Department of Pharmacognosy, Sivas Cumhuriyet University, Sivas, Turkey; 3 Facultad de Medicina, Universidad del Azuay, Cuenca, Ecuador; 4 University of Life Sciences “King Mihai I” from Timisoara, 300645 Calea Aradului 119, Timis, Romania; 5 Department of Microbiology, Victor Babes University of Medicine and Pharmacy of Timisoara, Timisoara, Romania; 6 Multidisciplinary Research Center on Antimicrobial Resistance, Timisoara, Romania; 7 Preventive Medicine Study Center, Timisoara, Romania

**Keywords:** Ursolic acid, Natural compound, Drug resistance, Cell signaling, Anti-cancer potential

## Abstract

Incidence rate of cancer is estimated to increase by 40% in 2030. Furthermore, the development of resistance against currently available treatment strategies has contributed to the cancer-associated mortality. Scientists are now looking for the solutions that could help prevent the disease occurrence and could provide a pain-free treatment alternative for cancers. Therefore, efforts are now put to find a potent natural compound that could sever this purpose. Ursolic acid (UA), a triterpene acid, has potential to inhibit the tumor progression and induce sensitization to conventional treatment drugs has been documented. Though, UA is a hydrophobic compound therefore it is usually chemically modified to increase its bioavailability prior to administration. However, a thorough literature indicating its mechanism of action and limitations for its use at clinical level was not reviewed. Therefore, the current study was designed to highlight the potential mechanism of UA, its anti-cancer properties, and potential applications as therapeutic compound. This endeavour is a valuable contribution in understanding the hurdles preventing the translation of its potential at clinical level and provides foundations to design new studies that could help enhance its bioavailability and anti-cancer potential for various cancers.

## Introduction

Cancer is a major public health issue and the second leading cause of death worldwide [1]. According to the Global Cancer Incidence, Mortality, and Prevalence (GLOBOCAN) report, about 19.2 million new cases of cancer and approximately 9.9 million cancer related deaths were reported in 2020. The burden of cancer is increasing and it has been estimated that by 2040 the number of cancer cases will reach up to 30 million [2]. Most treatment options used for cancer are chemotherapy in combination with surgical removal of cancer, radiation- and hormonal- therapy. The success of these strategies is limited by drug resistance, nonspecific targeting, and drug toxicity.

Researchers worldwide have been driven to evaluate the anticancer effect of biomolecules derived from natural sources due to the lack of effective chemo-preventive methods that are ideal for improving the therapeutic outcomes during an anticancer treatment. Owing to their enhanced safety, phytochemicals obtained from natural sources have the potential to be effectively used as a potent therapeutic agents against cancers [3]. One such active compound is UA (UA, 3-β-hydroxy-urs-12-en-28-oic acid) which is a triterpene acid commonly found in a variety of fruits, vegetables, and medicinal herbs [4, 5]. UA has been documented to possess the antioxidant [6], antidiabetic [7], and anti-inflammatory [8] properties. The anticancer potential of UA was also investigated in the studies conducted in the past decade [9]. Evidence showed that UA have the potential to promote cancer cell apoptosis [10], prevent angiogenesis [11, 12], and inhibit drug resistance [13].

Therefore, in current study, the detailed review of UA therapeutic potential in different cancers is provided that highlighted its role as chemo-preventive and treatment compound for the cancer. Similarly, the mechanism of action of UA in cancers is also brought to limelight that also facilitated in the identification of disease pathogenesis. This endeavour provided significant insight of UA potential for treatment of cancers in combination with conventional treatments.

### General characterization of UA

**Fig. 1:**
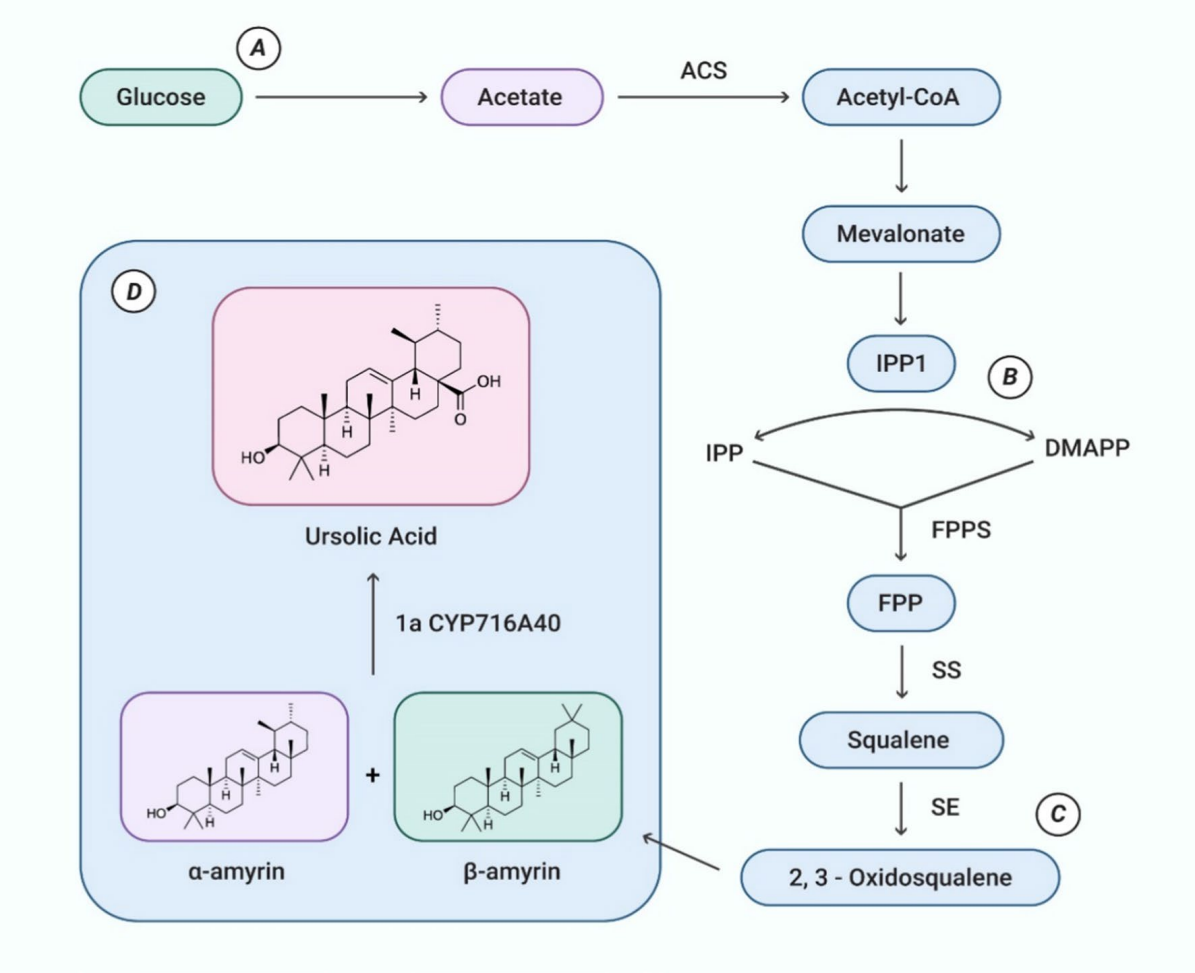
Synthesis of ursolic acid. **a** The process of ursolic acid synthesis begins with the conversion of glucose into acetate. Acetate is eventually converted into acetyl-CoA and mevalonate. **b** Several intermediate metabolites are produced in the process, mediated by several enzymes. **c** Squalene epoxidase (SE) finally produces 2,3-oxidosqualene that is converted into **d** α-amyrin and β-amyrin. Enzyme 1aCYP716A40 then produce ursolic acid from α-amyrin

Ursolic acid (UA), a common secondary metabolite, belongs to ursane-type pentacyclic triterpenoids, is found in the pomace, cork, flower, sprout, leaf, and bark of different medicinal plants [14]. UA was first extracted from apple waxes [15]. It’s percent composition varies from specie-to-specie due to the variation in abundance of the enzyme responsible for its synthesis [15]. It is a 3β-hydroxy-urs-12-en-28-oic acid with molecular formula C_30_H_48_O_3_, molecular weight 456.7 g/mol, and melting point of 283–285°C [16]. Owing to its low polarity, UA is poorly soluble in water, however, it shows high solubility in alcoholic sodium hydroxide and glacial acetic acid [17]. The biosynthesis of UA starts in plant cells by the folding and cyclization of squalene into (3S)-oxidosqualene which can result in the formation of 80 different carbon backbones [18]. The enzyme oxidosqualene cyclase is responsible for the carbocation rearrangement which produces this biological variation of carbon skeletons. The (3S)-oxidosqualene is a common precursor, gets converted to dammarenyl ring, forming the fifth ring present in lupeol, a-amyrin, and b-amyrin skeletons after going through ring expansion. The a-amyrin represents the UA skeleton [19]. The synthetic process for UA is illustrated in Fig. 1.

### Description of plant and plant sources

UA is a five-ring triterpenoid compound present in many botanical sources [20–23]. Family Lamiaceae is a well-known source of triterpenes. The leaves *Rosmarinus officinalis* are known as the commercial source of UA and contains its contents up to 2.95%. Recently, UA has been detected in commercially available dry fruits and edible wild-mushrooms for the first time [24, 25]. Different sources of UA are enlisted in Table 1.

**Table 1:** Botanical sources of UA

Source	References
*O. sanctum*	[26, 27]
*O. europaea*	[28]
*O. vulgare*	[28]
*R. officinalis*	[29]
*S. angustifolia*	[30]
*S. cordata*	[30]
*S. chirata*	[30]
*S. paniculata*	[30]
*P. flos*	[31]
*P. granatum*	[32]
*C. songaricum*	[33]
*L. camara*	[34]
*S. nigra*	[35]

Cuticular wax on leaves and fruits are also tremendous source of UA which protect the fruits from herbivore and bacterial, and mechanical stress [25]. Leaves and fruits of argan plants are also an important reservoir of UA. The by-products of agriculture industrious can serve as an beneficial alternative sources of UA [36]. High contents of UA can be found in fruits and peels of apples and persimmon fruits which are the waste products of juice industry. Further, several over-ripe fruits, such as cranberries, elderberries, bilberries, are also rich source of this triterpene acid [37].

UA has also been extracted from leftover of rosemary after carnosic acid extraction [38]. Moreover, the barks of *eucalyptus* trees can also be different source of UA [39]. The contents of UA in the plants can alter due to development stages of plant, environmental conditions, and seasonal variations. The contents of UA were found to be increased with the ripening in olive tree cultivars, and jujube plant [24]. Several different cultivars of grapes native to the northern European region have high contents of UA along with other triterpenoic acids and the levels of UAs were higher in fully developed mature fruits with contents up to 34–49% [40].

Studies have showed that the contents UA from different sources differ in a significant manner. For instance, the fruits and leaves of Vaccinium species have highest contents of free UA compared to the lower stems, roots and rhizomes, that contain bound form of UA [41]. Further, the altered geographical location of sources also affects the levels of UA. As the outer bark of *eucalyptus* trees present in the Temperate and the Mediterranean regions have higher concentrations of UA compared to the *Eucalyptus* trees present in the tropical and sub-tropical locations [24, 42].

### Semi-synthetic derivatives

The therapeutic potential of UA is hindered by its limited solubility, bioavailability, and fast metabolism. The poor solubility of UA in water results in low absorption, and short half-life of drug in the body. These obstacles limit the pharmacological applications of UA, and it has been placed in Class IV in biological drug classification system [43]. Researches have made various alteration using synthetic and semisynthetic approaches in the structure of UA to enhance its solubility in water and improve its pharmacological effects especially its anticancer properties [44]. Most of the chemical alterations in UA backbone are focused on positions C-3, C12-C13, and C-28 containing alcohol group, unsaturated double bond or alkene and carboxylic moiety respectively [45, 46]. Other groups of UA has also been modified which are categorized as miscellaneous modifications. Some of the recent studies involving the synthesis of UA derivatives to enhance its anticancer potential are discussed below (Table 2).

**Table 2:** List of UA derivates having anti-cancer activity

Derivatives	Modification site	Anticancer activity	Cell line	References
UA1-UA8	C-28	Antiproliferative activity	MCF-7, Hela, and A549	[11]
UA11b, UA7b	C-28	Cell cycle arrest, HIF1- α expression	SMMC-7721 and HepG2	[12]
FZU3010	C-28	Proliferation inhibition, Induction of apoptosis	SUM149PT, HCC1937	[14]
UA1a-2c	C-3	Apoptosis, Cell cycle arrest	VERO, HepG2	[16]
UA232	C-3, C-28	Induction of apoptosis	A549, H460, MCF-7, HeLa	[17, 18]

Tian and his group studied the anticancer activities of UA derivatives containing diamine moieties at C-28 on three different human cancer cell lines using MTT assay. The nitrogen containing UA derivatives were primarily synthesized by esterification at position C-28 using 2-hydroxyacetic acid. This C-28 modification was then followed by amidation reaction using amines including piperazine, *N*-methylpiperazine, alkane-1, 6-diamines, alkane-1, 4-diamines and alkane-1, and 2-diamines. The study showed that the derivatives of UA bearing primary amines showed more antiproliferative activities than that of derivatives with secondary and tertiary amines [47].

UA derivatives bearing oxidazole, triazolone, and piperazone moieties were synthesized and their antitumor potential was evaluated. The result revealed that compound 11b inhibited HIF1- α expression and enhanced antiproliferative activity by blocking the progression of cell cycle at G1 phase [48]. Similarly, another series of UA derivative containing an aminoguanidine moity were designed to inhibit HIF1-α expression. The compound 7b was found to be a promising HIF1-a inhibitor as it significantly inhibited the transcriptional activity of HIF1- α which was evaluated using luciferase reporter assay [49].

To enhance the bioavailability and anticancer activities, UA was chemically modified by Li et al. Using a nitrogenous heterocyclic scaffold and a privileged fragment at C-28, they synthesized a new derivative FZU3010. The results of Sulforhodimine B assay and flow cytometry showed that FZU3010 inhibited proliferation and induced apoptosis in SUM149PT and HCC1937 cell lines [50].

To enhance the hydrophilicity of UA to increase its clinical utility a simple synthetic approach was used to synthesize a series of new ionic UA derivatives. The results revealed a higher water solubility and improved anticancer activities of these novel UA ionic derivatives [51].

A total of six novel derivatives were synthesized through inducing modifications at C-3 position of UA. The UA derivative bearing amine moity at position C-3 showed the highest activity against leukemia cells. Moreover, the 2c showed high selectivity index for cancerous cells over normal cells. The mechanism of anticancer activities of 2c was studied and the results showed that the 2c derivative induces apoptosis through caspase 3, and 8; and causes cell cycle arrest. Furthermore, the synergistic effects of 2c and imatinib, which is a standard kinase inhibitor, was found when used in combination [52].

Shao et al. attempted to modify UA at C-3 and C-28 positions to synthesize a total of twenty-three derivatives with improved anticancer activities. They first performed acetylation of UA, resulting in the formation of 3-O-acetylursolic which was further treated with bromo-diolefine to obtain fatty esters. They also synthesized amides and ester derivatives of UA. The anticancer properties of UA derivatives were evaluated using MTT assay which revealed improved antiproliferative activities against the control group [53].

Wenfeng and colleagues prepared a series of UA derivatives and compared their anticancer activities with UA. They found that UA232 a novel derivative of UA possesses significantly higher anticancer activities against A549 and H460 cells than that of UA. Further expression analysis and flow cytometry revealed that UA232 induce G0/G1 phase arrest and apoptosis in lung cancer cell lines through endoplasmic reticulum mediated stress pathway [54]. Similarly, in a recent study UA232 inhibited proliferation and induced apoptosis in MCF-7 and HeLa cell lines. The observation of morphology of cells and western blot analysis revealed that UA232 also induce the biogenesis and membrane permeabilization of lysosomes [53].

### Mechanism of antitumor action of UA

**Fig. 2:**
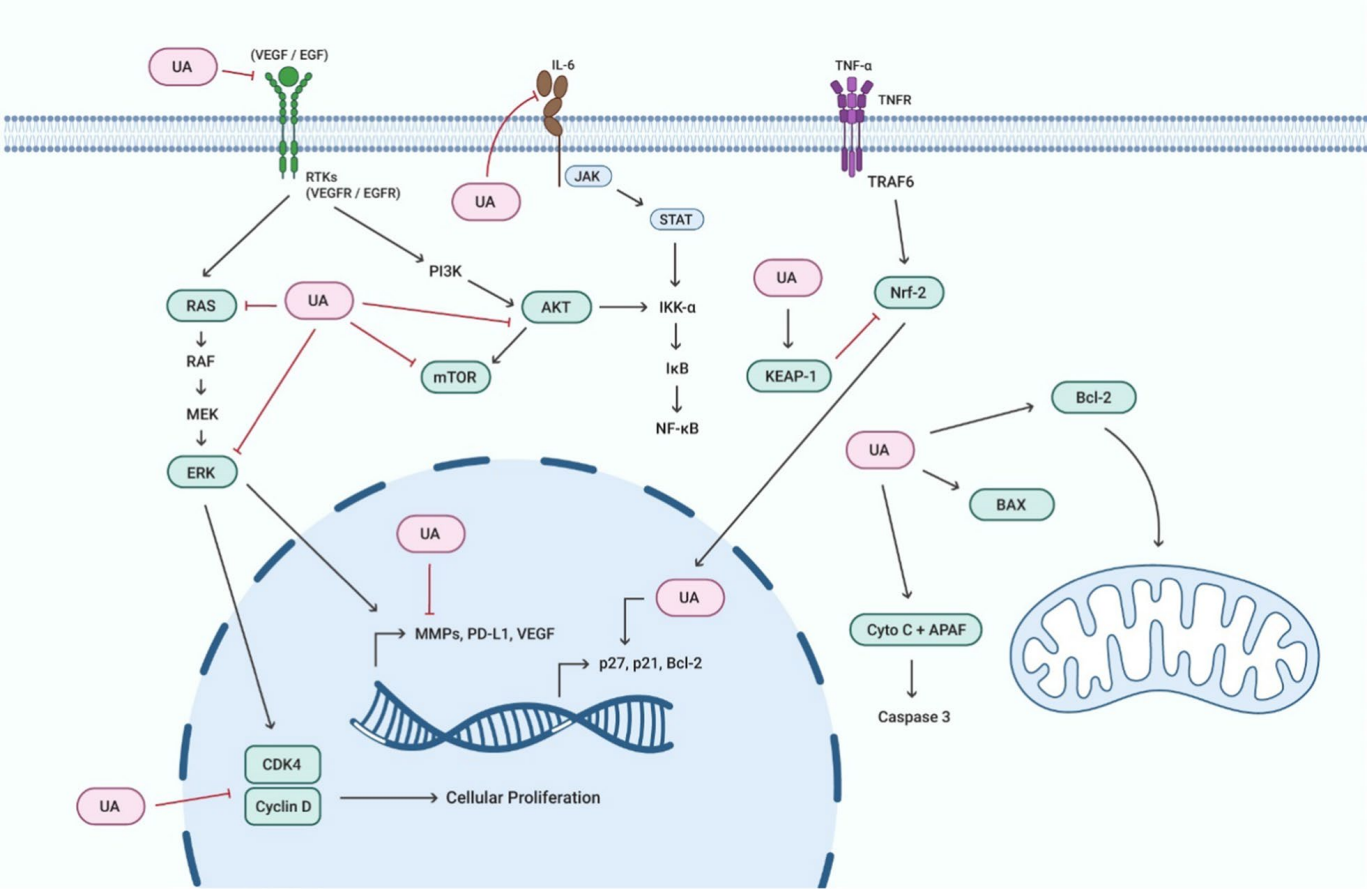
Mechanism of action of Ursolic acid in cancers. Ursolic acid targets numerous molecular players of different cell signaling cascades to promote its anti-cancer potential. It inhibits the activity of Akt, mToR, and ERK, and halts the receptor tyrosine kinase interaction with their respective ligands. It promotes the functioning of Bcl2, BAX, Cytochrome C, and KEAP1. It down-regulates the transcription of MMPs, PD-L1, and VEGF and up-regulates the expression of P27, P21, and Bcl2

UA has been under study for its various therapeutic effects in multiple diseases particularly in various types of cancers [55]. Investigations have revealed that UA targets multiple tumorigenic pathways and inhibits the abnormal cellular proliferation as well as the cancer cell metastasis in breast cancer (BC), colorectal cancer (CRC), ovarian Cancer (OC), lungs cancer (LC), and prostate cancer (PC) [9]. Various studies have demonstrated that UA performs its anti-cancer activity by targeting various cellular signalling pathways (Fig. 2).

#### MAPK pathway

In 2019, Jin et al. were successfully able to synthesize UA derivatives bearing moieties like thiadiazol, hydrazide, and oxadiazole and these compounds were studied on different cancer`s cell lines. One of the compounds, 4d was able to induce apoptosis in HeLa cells, and arrested the cell cycle at G0/G1 phase. Interestingly, this compound was also found to inhibit the MAPK pathway, as it was directly inhibiting the MEK1 activity, consequentially obstructing the RAS/RAF/MEK/ERK signal transduction pathway [56]. In another study, the melanoma cells of human were treated with UA which resulted in the inhibition of cancer cell growth and proliferation. The study showed that UA resulted in significant diminution in the expression of P38, and ERK proteins [57]. Effects of UA on MAPK signaling pathway in MG-63 cell showed that UA was significantly associated with induction of apoptosis by targeting ERK1/2 of MAPK pathway [58]. The anti-MAPK role of ursolic in BC was studied which showed that UA can inhibit the phosphorylation of RAF kinase, which are highly activated in BC, resulting in the inhibition of RAF/ERK pathway in the MCF-7 cell line [59]. In an In silico analysis, 113 protein targets of UA were identified and IL6, MAPK were the most prominent and major targeted pathways [60]. UA is also known to cause the inhibition of cell surface receptors including EGFR and other RTKs, that ultimately leads to the inactivation of downstream MAPK pathway, by preventing subsequent phosphorylation [61].

#### NF-KB pathway

UA can also effectively inhibit the proliferation and EMT in the gastric cancer cells via regulating the IKK/NF-KB pathway [62]. The study showed that when BGC cells were treated with UA, it resulted in decreased levels of IKK and NF-KB expression [63]. In 2015, Moser et al., showed that UA was inducing apoptosis in thyroid carcinoma cells by significantly increasing the activity of caspases and also resulted in the inhibition of IKK complex, ultimately resulting in NF-KB pathway activation [64]. UA was also found to have an inhibitory role on NF-KB pathway via the modification of IKKb residues [65]. Moreover, UA has also decreased carcinogenesis through the inhibition of IKBα and by phosphorylating and degrading IKK [66]. UA treatment of BC cell line significantly decreased the phosphorylated IKKβ, suggesting that UA is an inhibitor of IKK/NF-KB pathway [59]. UA inhibit the cell cycle progression of the tumor cells by halting them at G0/G1 phase by inactivating the IKK/NF-KB pathway. It also inhibit the activation of proinflammatory cytokines, resulting in inhibition of IKKα expression, leading towards phosphorylation inhibition of NF-KB [67].

#### JAK/STAT pathway

UA plays an essential role in the inhibition of JAK/STAT pathway. This compound inhibits the activation of src and JAK kinases by suppressing the phosphorylation, preventing the STAT3 activation in prostate cancer cells. Furthermore, UA also inhibited the xenografted prostate cancer in mouse model by inhibiting the activation of STAT3 [68]. UA also inhibits the colorectal cancer by inducing the apoptosis. Studies have showed that this compound can significantly lower the activation levels of JAK2 protein, which are anti-apoptotic in nature and further diminish the activation levels of STAT3 proteins, and further inhibiting their trans-localization towards the nucleus, thus preventing the transcriptional activation of anti-apoptotic genes in the colorectal cancer [69, 70]. Molecular mechanism underlying the UA activity was found to be the inhibition of jak-stat pathway and downregulation of VEGF, PD-L1, and MMPs. UA attaches to EGFRs and decrease the number of phosphorylated EGFRs in cell which were responsible for activation of Jak/Stat pathway. Products from jak/stat pathway were responsible for the expression of VEGF, MMPs and PD-L1 all of which are mediator of cell proliferation, Angiogenesis, and metastasis [71]. In hepatocellular carcinoma, UA is associated with the phosphorylation inhibition of JAK2 and STAT3. The study showed that that UA selectively inhibited the INF-a induced STAT3 in Hep3 cell line. UA also inhibited the activation of nuclear STAT. Furthermore, the treatment with UA also resulted in the elevated expression levels of pro-apoptotic genes, resulting in the suppression of tumor proliferation in in vivo settings [72]. UA is also affective against embryonic cancer stem cells. UA cause cell cycle arrest in ECSC increasing expression of P27 and P21 and decreasing expression of CDK4 and Cyclin D1 and it also cause apoptosis using BAX, Cytochrome C, BCL-xL, and BCL2 proteins. UA also increase the production of ROS that lead to DNA and cell damage [73].

#### PI3K/Akt/mTOR pathway

UA is also being used against ovarian cancer. In the current investigation, UA was used to treat ovarian cancer cells, and the inhibitory effects were clearly seen. By suppressing the activity of both PI3K and AKT molecules as well as their phosphorylated versions, UA blocked the PI3k/AKT pathway. Apoptosis and cell cycle arrest are caused by this as well as the elevated reactive oxygen species [74]. In case of oesophageal cancer cells, the UA shows anti-tumor activity mainly by causing autophagy. UA treated cells show a higher number of LC3 and lower number of P62 both are autophagy mediators. The mTOR initiated PI3k/AKT pathway is crucial in the autophagy inhibition. UA downregulates mTOR as well as downstream pathway that leads to the initiation of autophagy. UA is also thought to increase the Reactive oxygen species that also leads to autophagy [75]. UA treated cells indicated higher expression of LC3 that is an indicator of autophagy. Western blot analysis also indicated the inhibition of mTOR signalling pathway that was inhibitor of autophagy ultimately leading to autophagy [76]. Another breast cancer study on inhibitory effects of UA targeted breast cancer stem like cells derived from MCF-7. This study also concluded a significant inhibition of growth of these BCS like cells in cell culture settings. Moreover, western blot analysis indicated that UA resulted in the down regulation of p-PI3K, p-AKT, and p-ERK in BCS like cells in a dosage dependent manner [77].

### Current medical applications—official treatment or traditional medicine

#### Antimicrobial role

Bacterial infections impose a huge risk on public health, including the development antibiotics resistant strains. Now public health officials are considering to use the naturally occurring products and their derivatives as the potential therapeutic options against the microbial pathogens [78]. Scientists have explored the antibacterial properties of UA against methicillin resistant *Staphylococcus aureus* (MRSA) [79]. Studies have shown that UA affected the translation, metabolism, and redox balance of the microbes, leading towards the microbial cell death. UA C-3 derivatives were shown to have increased microbicidal activity against *Bacillus*, *Klebsiella*, *Escherichia*, *Shigella* species. Furthermore, when these derivatives were combined with kanamycin, their antimicrobial activity was enhanced and their minimum inhibitory concentration was also reduced to 8 μg/mL from 128 μg/mL [80].

Complexes of UA with metals such as copper, iron, zinc, antimony, and tin were shown to have antibacterial activity against both gram-positive and gram-negative microbes. The dimethyl tin derivative of UA was found to have excellent antibacterial activity with the MIC value of 8 μg/mL. However, the metal derivatives of copper and zinc did not demonstrated any activity in this regard [80–82]. In 2018, Zhao et al*.* extracted UA from leaves of *Ilex hainanensis* and form their derivatives to assess their activity against both gram-positive and gram-negative microbes, which showed that those compounds showed a significant levels of activity against gram-positive microbes, however their activity against gram-negative microbes was trivial [83]. The extracts of UA from diospyros leaves were investigated for their antimicrobial activity where they were shown to inhibit the biofilm formation by *E. coli* for 24 h at the concentration of 10 μg/mL [84]. In another study, two of the UA derivatives were shown to have high cytotoxic and cysticidal activity against *Acanthamoeba* spp. demonstrating the role as amoebicidal drug [85]. In the recent pandemic of Covid-19, researchers have identified the potential therapeutic role of UA. It has been postulated that this compound can inhibit the protease of SARS-CoV-2, ultimately inhibiting its entry to the cells. Furthermore, UA diminishes oxidative stress and inflammatory response induced by the viral infection [86, 87].

Several modern studies have implied that the excessive use of antimicrobial agents, during and after the treatment of cancer can also cause undesirable consequences. As it is now known that antimicrobial agents have adverse effect on human gut microbiota, which can persist for massive period of time [88]. This can result in gut dysbacteriosis, causing disbalance between host and gut microbiome [89]. Therefore, antimicrobial agents do not only results in the disruption of gut microbiota, but also stimulate inflammatory responses and reduced immune response, that subsequently hampers the efficacy of the cancer treatment [90]. Though, the double-edged role of UA as antimicrobial agent in treatment of cancer patients has not yet been explored yet but such study can be conducted in future to analyze the impacts of antimicrobial function of UA on cancer patients and to modify its properties to provide benefits to the cancer patients.

#### Neuroprotectant role

Inflammation in the neuron can lead towards serious neurological disorders which can dangerously impact an individual’s health. When any neurotoxin, pathogen or indigenous source cause the microglial cells to activate, it results in the activation of inflammatory response mediated by prostaglandins, ROS, NO, cytokines, chemokines, ultimately leading towards neurodegenerative diseases [81, 91, 92]. Various studies have showed that UA can play an essential neuromodulatory function [93]. In 2019, study showed that when the Parkinson’s mouse models were treated with UA (25 mg/kg body weight) it resulted in the regression of inflammatory response by targeting the NF-κB pathway [94]. The antidepressant role of UA is under investigation. Studies have showed that anti-oxidant activity of UA is essential for the treatment of depression [95]. Further in vivo analysis have revealed that UA upregulate the expression of Nrf-2 and Prdx-2 genes which have neuroprotective role and protect the nematode *C. elegans* from the injurious effects of reactive oxygen species [96].

UA extract from *P. incarnata* was shown to have a neuroprotective role Alzheimer’s disease which is characterize by the accumulation of amyloid-β plagues. When Aβ-induced mice model were administered with UA (56 mg/kg), there was significant decrease in the accumulation of the amyloid plagues with the improvement in performance of spatial memory [97]. In another study the neuroprotective role of UA was studied in *C. elegens* which showed that at the concentration of 100 μM, UA significantly inhibited the amyloid-β by augmenting the ubiquitin proteosome system which is essential for the maintenance of proteostasis [98]. Protective role of UA against oxidative neural injury have also been studied. The studies elucidated that when rat brain tissues were depleted of antioxidant enzyme system it resulted in increased oxidative stress. However, when brain tissues were treated with UA the levels and activities of antioxidant enzymes became normal suggesting the neuroprotective potential of triterpene acid [93, 99].

#### Anti-diabetic and anti-obesity role

Increased global incidence of obesity and diabetes have inflicted a tremendous risk to the well-being of human. Diabetes mellitus is characterize by the deficiency and resistance to insulin, resulting in hyperglycemia, moreover obesity has a strong association with diabetes and insulin resistance [100–103]. This condition can lead towards various health conditions including cardiovascular diseases, neuropathy, nephropathy, retinopathy [104–106]. Therefore, there is an urgent need to identify such natural products that can cure this disease [9, 107]. Studies have shown that UA can be a possible therapeutic impact in reducing hyperglycemia and treating the diabetes-related problems in hyperglycemic mice models [108]. Moreover, the UA derivatives are also revealed to reduce the levels of tyrosine phosphatase, resulting in enhanced phosphorylation of insulin receptors, causing the absorption of glucose [109, 110]. In a recent study when the high-fat diet (HFD) and hyperglycemic mice were treated with UA (10 mg/kg) for 8 weeks, their blood glucose levels were significantly decreased. Moreover, the levels of their plasma insulin, blood triglyceride and cholesterol, and oxidative species were also alleviated. The UA was also found to have inhibitory role in Mapk-8, JNK, and insulin pathways and boosted the regeneration of insulin producing β-cells of pancreas [111].

The bioactive activity of UA extracts from asparagus wild was observed which showed that UA inhibit the activity of two key metabolic enzymes α-glucosidase and α-amylase which are involved in type II diabetes [112]. In a study with mice models, diabetes was induced through HFD and streptozotocin. Administration of UA was found to have a beneficial role diabetes-induced nephropathy [113]. Furthermore, in another study, when diabetes-mice models were treated with UA there was a downregulation of TNF-α, MMP-2 and improved the condition of cardiomyopathy [114]. Hence, various studies have proved that treatment with UA is advantageous for the treatment of diabetes and consequent problems in liver, kidney, muscles and heart [115].

#### Anti-inflammatory role

Inflammation is a response of body against pathogenesis induced by any harmful agent or microbes to maintain tissue homeostasis. However, when the inflammatory response is prolonged and become chronic, it causes damage to the tissues and malfunctioning of the organs [116, 117]. Chronic inflammation then forms a baseline for various diseases including neurodegenerative disorders, hepatic, renal, pulmonary, and cardiovascular diseases. It is now known that inflammation is associated with increased cytokine activity and elevated levels of ROS, promoting cancer [9, 12]. The studies have shown that UA and its derivatives have tendency to diminish the levels of Tnf-α, NF-κB and other inflammatory proteins, lowering the inflammation and oxidative stress in cell lines infected with *M. tuberculosis* [37]. Zhou et al. investigated the role of UA extracted from Chinese traditional-medicine plants on RAW264 7 cell and it was shown that UA reduced the expression of NF-κB, Tnf-α, Cox-2, decreased localization of NF-κB to nucleus, and inhabited the production of NO [118]. UA has been also discovered to play protective role in inflammation-induced acute kidney injury (AKI). In vitro cell culture analysis showed that UA significantly reduced the levels of inflammatory cytokines IL-6, IL-1β. Moreover, UA also inhibited the inflammatory pathway mediated by Tlr4/Myd88 and directed the macrophages to autophagy by activation of LC3 B, and Beclin1 [119].

Further, the UA derivates (1,2,3-trizole) were designed and accessed for their anti-inflammatory activity. It was depicted that the compounds were inhibiting the Cox-1 and Cox-2 enzymes and reducing the inflammation by the inhibition of prostaglandins biosynthesis [120, 121]. The inhibition of Cox-1, Cox-2, IL-1, and Tnf-α ameliorated the arthritis in animal models [122]. In 2020, Wang et al. analyzed the protective role of UA in inflammation of chondrocytes and relieve osteoarthritis (OA). UA was found to downregulate the expression of pro-inflammatory cytokines, PTGS2, MMP13, NLRP3, and inhibit the NF-Κb/NLRP inflammasome, prevent the inflammation and degeneration of cartilage and ameliorate the osteoarthritis [123].

#### Scientific studies confirmed the anticancer properties

**Fig. 3:**
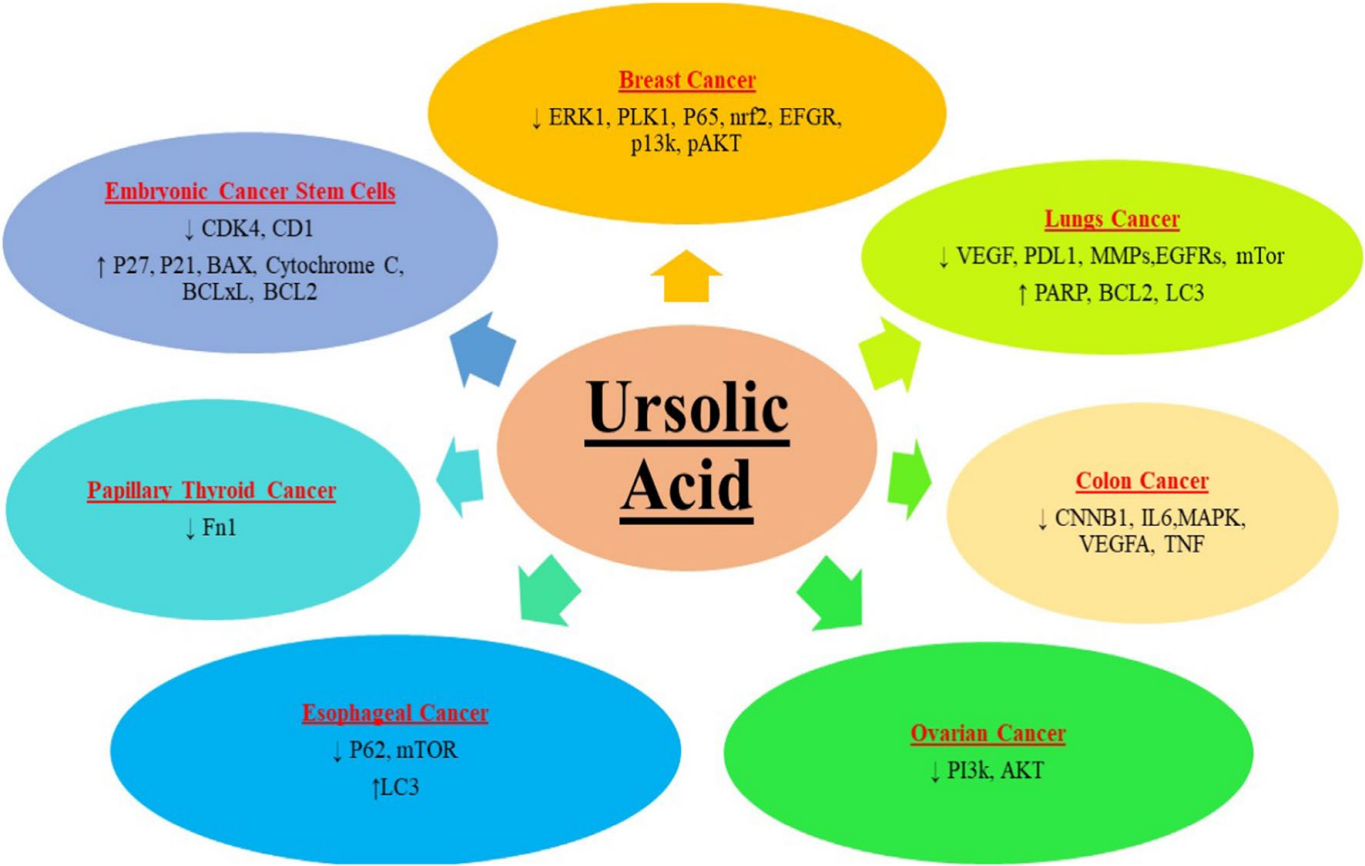
Different targets of ursolic acid in different cancers. Arrow pointing down indicates down-regulation or decrease in activity and arrow pointing up represents increased activity or up-regulation

Numerous studies have confirmed the anticancer properties of UA in vitro. However, its mechanism of action varies from cancer to cancer. The molecular players targeted by UA in different cancers is illustrated in Fig. 3.

#### Breast cancer

UA (UA) shows potent anti-cancer properties against breast cancer (BC) cells by inducing apoptosis and autophagy, cell cycle and cell proliferation arrest and suppressing angiogenesis and metastasis. [12]. It was observed that UA induced autophagy in BC cell lines via endoplasmic reticulum stress by up-regulating myeloid cell leukemia sequence 1 and mitogen-activated protein kinase (MAPK) [12, 124]. The migratory property of BC cells was suppressed due to the administration of UA in addition to induction of apoptosis due to up-regulation in the glycogen synthase kinase activity followed by down-regulation of B-cell lymphoma 2. It was also observed that UA inhibited inflammation in BC cells by down-regulating NF-κB, thus halting further progression [37]. A synthetic derivative of UA, FZU3010 caused cell cycle arrest in BC cells at S and G0/G1 phase leading to programmed cell death [50].

The chemo-preventive effect of UA was also elucidated in metastatic BC cell lines through inactivation of Jun N-terminal kinase, Akt and mammalian target of rapamycin phosphorylation and lowering NF-κB levels in the nucleus [50, 125]. In another study it was observed that Bcl-2 down-regulation and Bax up-regulation was caused by UA, resulting in release of cyt-c from mitochondrial membrane into cytoplasm. UA can cleave Caspase-9 and decreased mitochondrial membrane activity in a death receptor dependent manner [126]. The anti-cancer properties of UA have also been observed in some in vivo models against BC cells. Modulation of Akt/mTOR signal pathway and induction of apoptosis was observed in post-menopausal mice injected with BC cells [12, 127]. In another study UA administered through folate-chitosan nanoparticles resulted in decreased MCF-7 xenograft in mice models [12, 128].

#### Colorectal cancer

Therapeutic potential of UA has also been studied in colorectal cancer (CRC) with positive results. STAT3 signal transduction pathway is involved in CRC progression and its inhibition has been linked with decreased cancer cell growth [129, 130]. UA inhibits STAT3 phosphorylation and reduces colon cancer-initiating cells’ viability. It was also observed that UA affected the tumor sphere forming ability thus halting tumor growth in CRC [12, 130]. HT-29 colon cancer cell line growth was inhibited through the inhibition of EGFR/MAPK pathway in addition to down-regulation of Bcl-2 and activation of caspase-3 and caspase-9 [100, 131]. In a recent study it was observed that UA enhanced the therapeutic activity of oxaliplatin in CRC cell lines through ROS-mediated inhibition of drug resistance genes, inhibition of proliferation and induction of apoptosis [13].

Effect of UA on CRC cells in mice models revealed that UA reduced tumor volume without affect body weight of mice, which indicates its anti-tumor activity without toxicity to normal body cells. US also halted the expression of angiogenic factors such as vascular endothelial growth factor A and basic fibroblast growth factor in addition to suppression of sonic hedgehog (SHH), STAT3, Akt and p70S6K pathways [11, 12]. In another study the underlying mechanism of apoptosis induction by UA were analyzed and it was found that UA induced cleavage of PARP and caspase-3 in HCT116 and HT29 cell lines. It also halted the phosphorylation of JAK2 leading to inhibition of nuclear localization of STAT3 [69]. It was also observed that UA administration to the HCT116 and HT29 cell lines up-regulated miR-4500, which is a known tumor suppressor in CRC via the inhibition of JAK/STAT3 pathway [69, 132].

#### Prostate cancer

Impact of UA in prostate cancer (PCa) has also been explored and it has been seen in a recent study that UA promotes apoptosis in PCa via ROCK/PTEN pathway. Rho associated protein kinase (ROCK) interacts with microtubules and is essential for maintenance of epithelial cell polarity [133]. UA inhibited ROCK protein expression and promotes the expression of cleaved ROCK expression in PCa cell lines. UA also promoted the PTEN up-regulation which also induces apoptosis in PCa cells [134, 135]. UV expressed cytotoxic effects against PC-3 cell line by cleaving PARP, activating caspase-9 and caspase-3, suppressing Bcl2, Mcl-1 and up-regulating Bax. It also lowered the expression of Wnt5α/β and β-catenin, and up-regulated the phosphorylation of glycogen synthase kinase 3 β (GSK3β), leading to cell death [134, 136]. In a recent it was observed that UA slowed tumor growth in mice transplanted with VCaP PCa cells via metabolic remodeling and epigenetic methylation reprogramming. Epigenetic reprogramming was achieved through up-regulation of mitochondrial metabolite S-Adenosyl methionine with increased UA administration, indicating its anti-cancer properties [137]. SETD7 (SET Domain Containing 7, Histone Lysine Methyltransferase) is involved in the maintenance of chromatin and contrls transcription of various genes. It is also involved in stabilizing Nrf2 protein, which is involved in protection from DNA damage and genotoxicity [138]. In a recent study it was observed that UA induced SETD7 expression in PC-3 cells and induced anti-oxidant and DNA protective effects through the Nrf2/ARE signaling pathway [139].

#### Pancreatic and liver cancer

Rising chemoresistance rates for prostate cancer cases has exacerbated the need to find safer treatment options. It was observed that UV induced anti-tumor effects trough increased ER stress and autophagy in pancreatic cancer cells. Levels of ER stress-related proteins such as CHOP, p-eIF2α, BiP, and Calpain1 were increased upon UA administration leading reduced cell viability. It was also seen that UA induced autophagy through ATG5/LC3 II-dependent signaling. Administration of UA with Gemcitabine (GEM) increased cell death and improved chemo-sensitivity to GEM in pancreatic cancer cells [140]. Role of UA in improving drug resistance in pancreatic cells was evaluated in another recent study, which showed that treatment with UA significantly lowered the levels of receptor for advanced glycation end products (RAGE), nuclear factor kappa B p65 (NF-κB/p65), and multidrug resistance protein 1 (MDR1) in pancreatic cancer cells. These proteins are elevated in GEM resistant tumor cells. It was further observed that simultaneous treatment with UA and GEM significantly decreased the RAGE/NF-κB/MDR1 cascade and halted tumor growth [141].

In case of hepatocellular carcinoma (HCC), up-regulated cholesterol and lipid metabolism promote tumor growth through stimulation of oncogenic signaling pathways [142]. Lowered cholesterol levels upon administration of UA in HCC cells was observed and it was proposed that this may be the mechanism of anti-cancer role of UA in HCC. It was observed that UA caused cell cycle arrest at the G0/G1 checkpoint due to insufficient cholesterol biosynthesis [105].

#### Metabolism of UA

In addition to having anti-cancerous properties, UA and its derivatives can also block G + and G– bacteria as well as a few viral actions [143]. UA has furthermore powerful diuretic, hypotensive, lipid-regulating, and hypoglycemic actions, according to reports. Consequently, UA has various pharmacological effects. The isolation, extraction, identification, and pharmacological effects of UA have been the subject of several investigations recently. The pharmacokinetic behaviour of UA, particularly its absorption and metabolism, has not been the subject of many investigations. The processing of UA in vivo may be significantly influenced by drug-metabolizing enzymes and ATP-binding cassette transporters (ABC transporters), such as P-glycoprotein (Pgp), multidrug resistance-associated proteins (MRPs), and breast cancer-resistant protein (BCRP). According to a research, the predominant mechanism for UA absorption in the intestinal tract is passive transport, however Pgp may also be engaged in active efflux transport [144]. UA is most likely the P-gp substrate that is connected to the efflux of the drug transporter. At the same time, a research employing human liver microsomes revealed that UA had inhibitory effects on CYP450 isoforms, suggesting that UA may be the substrate of CYP450 [145]. Therefore, the metabolism of UA may include CYP450. The pregnane X receptor (PXR) is crucial in controlling the expression of a few metabolic enzymes and important drug transporters. PXR is crucial to the pharmacokinetics and pharmacodynamics of medications [146]. Researchers looked at the PXR’s function in UA metabolism since ligands can specifically bind to it in various species. They discovered that Pgp, BCRP, and MRP2—three ABC drug transporters—were involved in the efflux of UA in the gut. The metabolism of UA involves both CYP3A4 and CYP2C9, and PXR-RXR could considerably boost CYP2C9 expression in the Caco2 cell line [147].

#### Potential studies

UA is a naturally occurring substance that has been used in a variety of ways due to its biological properties and demonstrates its potential to be effective in the treatment and prevention of several diseases. As a result, in both cellular and animal models, the pharmacological effects of UA demonstrated anti-cancer, anti-diabetic, neuroprotective, and anti-inflammatory mechanisms. Despite the great advances that have been made in elucidating the role of UA in various pathologies and its pharmacological properties, there are still gaps in the research that need to be filled. UA is a multi-dimensional molecule that targets many different pathways within cells by regulating transcription factors, protein kinases and metabolites etc. One of the major concerns regarding UA is its poor bioavailability and low solubility and intestinal permeability despite its strong pharmacological properties [148]. UA is classified as BCS class IV compound according to Biopharmaceutical Classification System (BCS) and needs nanotechnology based drug delivery system [149]. The pharmacological properties of UA will be greatly altered depending upon the type and composition of the carrier nanoparticle including particle size, potential charges and biomimetic properties. In case of breast cancer UA nanoparticle delivery and its anti-cancer properties have been observed with positive results [150], however each type of cancer has its unique biological properties and various pathways are modulated. In vitro studies of various types of UA nanoparticle drug delivery systems are required to check their therapeutic potential, mechanisms of action, bioavailability, and cytotoxicity. In addition, in vivo models are required for the in depth analysis of the route of administration, dose and frequency of drug administration to evaluate the physicochemical properties and pharmacokinetic profile of the drug.

By structurally altering the original UA skeleton, researchers have produced synthetic UA derivatives with improved medicinal benefits to overcome the problems of poor bioavailability and intestinal uptake [151]. When compared to UA, these structurally changed molecules have improved therapeutic benefits. The C-2 position, -hydroxyl (C-3), and carboxylic moieties (C-28) of UA were the main locations for the change of its structure, according to several studies on pentacyclic triterpenoids. The majority of studies improved the chemical or physical activities of the UA molecule by altering the molecular structure of UA around the three spots [9]. However, hybridization of UA based compounds with known chemotherapeutic molecules to determine its bioavailability and toxicology profile has not been done yet and can be explored in the future.

## Conclusion

A natural compound, ursolic acid, can be isolated from several commonly consumed fruits and vegetables. Its ability to simultaneously target multiple signalling proteins has capacitated it to be used as chemo preventive or therapeutic agent for cancers. It inhibits or promotes the activity of numerous proteins belonging to cell growth and cell apoptosis, respectively. Despite the availability of the tons of data, there is a need to perform more proteomics-based research to understand its potential as therapeutic agent. Similarly, the impact of UA on the metabolism of a cancer cell must also be investigated. There is also a need to integrate nanotechnology to enhance the bioavailability and anti-cancer potential of UA. Studies at cell level has shown the promising outcomes. Therefore, future investigations must be focused on evaluating its cytotoxicity in animal models and determining its pharmacological dose in different cancers. Through more investigations UA can be delineated as pain-free therapeutic option for cancers.

## Data Availability

Not Applicable.

## References

[1] Oh CM, Lee D, Kong HJ, Lee S, Won YJ, Jung KW, Cho H (2020). Causes of death among cancer patients in the era of cancer survivorship in Korea: attention to the suicide and cardiovascular mortality. Cancer Med.

[2] Ferlay J, Ervik M, Lam F, Colombet M, Mery L, Piñeros M, Znaor A, Soerjomataram I, Bray F (2020). Global cancer observatory: cancer today.

[3] Khwaza V, Oyedeji OO, Aderibigbe BA (2020). Ursolic acid-based derivatives as potential anti-cancer agents: an update. Int J Mol Sci.

[4] Alam M, Ali S, Ahmed S, Elasbali AM, Adnan M, Islam A, Hassan MI, Yadav DK (2021). Therapeutic potential of ursolic acid in cancer and diabetic neuropathy diseases. Int J Mol Sci.

[5] Yin R, Li T, Tian JX, Xi P, Liu RH (2018). Ursolic acid, a potential anticancer compound for breast cancer therapy. Crit Rev Food Sci Nutr.

[6] Liobikas J, Majiene D, Trumbeckaite S, Kursvietiene L, Masteikova R, Kopustinskiene DM, Savickas A, Bernatoniene J (2011). Uncoupling and antioxidant effects of ursolic acid in isolated rat heart mitochondria. J Nat Prod.

[7] Yu S-G, Zhang C-J, Xu X-E, Sun J-H, Zhang L, Yu P-F (2015). Ursolic acid derivative ameliorates streptozotocin-induced diabestic bone deleterious effects in mice. Int J Clin Exp Pathol.

[8] Kashyap D, Sharma A, Tuli HS, Punia S, Sharma AK (2016). Ursolic acid and oleanolic acid: pentacyclic terpenoids with promising anti-inflammatory activities. Recent Pat Inflamm Allergy Drug Discov.

[9] Khwaza V, Oyedeji OO, Aderibigbe BA (2020). Ursolic acid-based derivatives as potential anti-cancer agents: an update. Int J Mol Sci.

[10] Zhang H, Li X, Ding J, Xu H, Dai X, Hou Z, Zhang K, Sun K, Sun W (2013). Delivery of ursolic acid (UA) in polymeric nanoparticles effectively promotes the apoptosis of gastric cancer cells through enhanced inhibition of cyclooxygenase 2 (COX-2). Int J Pharm.

[11] Lin J, Chen Y, Wei L, Hong Z, Sferra TJ, Peng J (2013). Ursolic acid inhibits colorectal cancer angiogenesis through suppression of multiple signaling pathways. Int J Oncol.

[12] Chan EWC, Soon CY, Tan JBL, Wong SK, Hui YW (2019). Ursolic acid: an overview on its cytotoxic activities against breast and colorectal cancer cells. J Integr Med.

[13] Zhang Y, Huang L, Shi H, Chen H, Tao J, Shen R, Wang T (2018). Ursolic acid enhances the therapeutic effects of oxaliplatin in colorectal cancer by inhibition of drug resistance. Cancer Sci.

[14] Jäger S, Trojan H, Kopp T, Laszczyk MN, Scheffler A (2009). Pentacyclic triterpene distribution in various plants–rich sources for a new group of multi-potent plant extracts. Molecules.

[15] Woźniak Ł, Skąpska S, Marszałek K (2015). Ursolic acid—a pentacyclic triterpenoid with a wide spectrum of pharmacological activities. Molecules.

[16] Son J, Lee SY (2020). Therapeutic potential of ursonic acid: comparison with ursolic acid. Biomolecules.

[17] Fan J-P, Kong T, Zhang L, Tong S, Tian Z-Y, Duan Y-H, Zhang X-H (2011). Solubilities of ursolic acid and oleanolic acid in four solvents from (283.2 to 329.7) K. J Chem Eng Data.

[18] Ebizuka Y, Katsube Y, Tsutsumi T, Kushiro T, Shibuya M (2003). Functional genomics approach to the study of triterpene biosynthesis. Pure Appl Chem.

[19] Babalola IT, Shode FO (2013). Ubiquitous ursolic acid: a potential pentacyclic triterpene natural product. J Pharm Phytochem.

[20] Baliga MS, Shivashankara AR, Venkatesh S, Bhat HP, Palatty PL, Bhandari G, Rao S (2019). Phytochemicals in the prevention of ethanol-induced hepatotoxicity: a revisit.

[21] Shivashankara AR, Venkatesh S, Bhat HP, Palatty PL, Baliga MS, Watson Ronald (2015). Can phytochemicals be effective in preventing ethanol-induced hepatotoxicity in the geriatric population? an evidence-based revisit. Foods and dietary supplements in the prevention and treatment of disease in older adults.

[22] Shi P, Zhang Z, Xu J, Zhang L, Cui H (2021). Endoplasmic reticulum stress-induced cell death as a potential mechanism for targeted therapy in glioblastoma. Int J Oncol.

[23] Jäger S, Trojan H, Kopp T, Laszczyk MN, Scheffler A (2009). Pentacyclic triterpene distribution in various plants–rich sources for a new group of multi-potent plant extracts. Molecules.

[24] López-Hortas L, Pérez-Larrán P, González-Muñoz MJ, Falqué E, Domínguez H (2018). Recent developments on the extraction and application of ursolic acid. Food Res Int.

[25] Ali SA, Ibrahim NA, Mohammed MM, El-Hawary S, Refaat EA (2019). The potential chemo preventive effect of ursolic acid isolated from Paulownia tomentosa, against N-diethylnitrosamine: initiated and promoted hepatocarcinogenesis. Heliyon.

[26] Pattanayak P, Behera P, Das D, Panda SK (2010). *Ocimum sanctum* Linn. a reservoir plant for therapeutic applications: an overview. Pharmacogn Rev.

[27] Singh D, Chaudhuri PK (2018). A review on phytochemical and pharmacological properties of Holy basil (*Ocimum sanctum* L.). Ind Crops Prod.

[28] Al-Tannak NF, Novotny L. 2020 LC-MS method for the detection and quantification of ursolic acid and uvaol levels in olive leaves and oregano. Emir J Food Agric. 600–609

[29] Li P, Liu A, Li Y, Yuan B, Xiao W, Liu Z, Zhang S, Lin H (2019). Development and validation of an analytical method based on HPLC-ELSD for the simultaneous determination of rosmarinic acid, carnosol, carnosic acid, oleanolic acid and ursolic acid in rosemary. Molecules.

[30] Boral D, Moktan S (2021). Predictive distribution modeling of *Swertia bimaculata* in darjeeling-sikkim Eastern Himalaya using MaxEnt: current and future scenarios. Ecol Process.

[31] Meng X, Liu D, Yang M, Shi Y, He H (2020). Establishment of extraction design space for ursolic acid from *Paulowniae Flos* based on the concept of quality by design. Phytochem Anal.

[32] Sun S, Huang S, Shi Y, Shao Y, Qiu J, Sedjoah R-CA-A, Yan Z, Ding L, Zou D, Xin Z (2021). Extraction, isolation, characterization and antimicrobial activities of non-extractable polyphenols from pomegranate peel. Food Chem.

[33] Cai C, Wu S, Wang C, Yang Y, Sun D, Li F, Tan Z (2019). Deep eutectic solvents used as adjuvants for improving the salting-out extraction of ursolic acid from *Cynomorium songaricum* Rupr. in aqueous two-phase system. Sep Purif Technol.

[34] Jamal M, Amir M, Ali Z, Mujeeb M (2018). A comparative study for the extraction methods and solvent selection for isolation, quantitative estimation and validation of ursolic acid in the leaves of *Lantana camara* by HPTLC method. Futur J Pharm Sci.

[35] Milena V, Tatjana M, Gökhan Z, Ivana B, Aleksandra C, Mohammad MF, Marija R (2019). Advantages of contemporary extraction techniques for the extraction of bioactive constituents from black elderberry (*Sambucus nigra *L.) flowers. Ind Crops Prod.

[36] Rubashvili I, Tsitsagi M, Zautashvili M, Chkhaidze M, Ebralidze K, Tsitsishvili V (2020). Extraction and analysis of oleanolic acid and ursolic acid from apple processing waste materials using ultrasound-assisted extraction technique combined with high performance liquid chromatography. Rev Roum Chim.

[37] Cargnin ST, Gnoatto SB (2017). Ursolic acid from apple pomace and traditional plants: a valuable triterpenoid with functional properties. Food Chem.

[38] Hilali S, Fabiano-Tixier A-S, Elmaataoui M, Petitcolas E, Hejjaj A, Aitnouh F, Idlimam A, Jacotet-Navarro M, Bily A, Mandi L (2018). Deodorization by solar steam distillation of rosemary leaves prior to solvent extraction of rosmarinic, carnosic, and ursolic acids. ACS Sustain Chem Eng.

[39] Gupta A, Maheta P, Chauhan R, Pandey S, Yadav JS, Shah S (2018). Simultaneous quantification of bioactive triterpene acids (ursolic acid and oleanolic acid) in different extracts of *Eucalyptus globulus* (L) by HPTLC method. Pharm J.

[40] Vujasinović V, Bjelica M, Čorbo S, Dimić S, Rabrenović B (2021). Characterization of the chemical and nutritive quality of cold-pressed grape seed oils produced in the Republic of Serbia from different red and white grape varieties. Grasas Aceites.

[41] Vilkickyte G, Raudone L (2021). Phenological and geographical effects on phenolic and triterpenoid content in *Vaccinium vitis-idaea *L leaves. Plants.

[42] Dheyab AS, Ibrahim AJK, Aljumily EK, AlOmar MK, Bakar MFA, Sabran SF (2022). Antimycobacterial activity and phytochemical properties of *Eucalyptus camaldulensis* (eucalyptus) extracted by deep eutectic solvents. Mater Today Proc.

[43] Yu D, Kan Z, Shan F, Zang J, Zhou J (2020). Triple strategies to improve oral bioavailability by fabricating coamorphous forms of ursolic acid with piperine: enhancing water-solubility, permeability, and inhibiting cytochrome p450 isozymes. Mol Pharm.

[44] Chen H, Gao Y, Wang A, Zhou X, Zheng Y, Zhou J (2015). Evolution in medicinal chemistry of ursolic acid derivatives as anticancer agents. Eur J Med Chem.

[45] Tu H-Y, Huang AM, Wei B-L, Gan K-H, Hour T-C, Yang S-C, Pu Y-S, Lin C-N (2009). Ursolic acid derivatives induce cell cycle arrest and apoptosis in NTUB1 cells associated with reactive oxygen species. Bioorg Med Chem.

[46] Hua S-X, Huang R-Z, Ye M-Y, Pan Y-M, Yao G-Y, Zhang Y, Wang H-S (2015). Design, synthesis and *in vitro* evaluation of novel ursolic acid derivatives as potential anticancer agents. Eur J Med Chem.

[47] Tian T, Liu X, Lee E-S, Sun J, Feng Z, Zhao L, Zhao C (2017). Synthesis of novel oleanolic acid and ursolic acid in C-28 position derivatives as potential anticancer agents. Arch Pharm Res.

[48] Chi K-Q, Wei Z-Y, Wang K-S, Wu J, Chen W-Q, Jin X-J, Piao H-R (2017). Design, synthesis, and evaluation of novel ursolic acid derivatives as HIF-1α inhibitors with anticancer potential. Bioorg Chem.

[49] Wu J, Zhang Z-H, Zhang L-H, Jin X-J, Ma J, Piao H-R (2019). Design, synthesis, and screening of novel ursolic acid derivatives as potential anti-cancer agents that target the HIF-1α pathway. Bioorg Med Chem Lett.

[50] Li W, Zhang H, Nie M, Wang W, Liu Z, Chen C, Chen H, Liu R, Baloch Z, Ma K (2018). A novel synthetic ursolic acid derivative inhibits growth and induces apoptosis in breast cancer cell lines. Oncol Lett.

[51] Fan J-P, Lai X-H, Zhang X-H, Yang L, Yuan T-T, Chen H-P, Liang X (2021). Synthesis and evaluation of the cancer cell growth inhibitory activity of the ionic derivatives of oleanolic acid and ursolic acid with improved solubility. J Mol Liq.

[52] da Silva EF, de Vargas AS, Willig JB, de Oliveira CB, Zimmer AR, Pilger DA, Buffon A, Gnoatto SCB (2021). Synthesis and antileukemic activity of an ursolic acid derivative: a potential co-drug in combination with imatinib. Chem-Biol Inter.

[53] Gou W, Luo N, Yu B, Wu H, Wu S, Tian C, Guo J, Ning H, Bi C, Wei H (2022). Ursolic acid derivative UA232 promotes tumor cell apoptosis by inducing endoplasmic reticulum stress and lysosomal dysfunction. Int J Biol Sci.

[54] Gou W, Luo N, Wei H, Wu H, Yu X, Duan Y, Bi C, Ning H, Hou W, Li Y (2020). Ursolic acid derivative UA232 evokes apoptosis of lung cancer cells induced by endoplasmic reticulum stress. Pharm Biol.

[55] Zheng H, Feng H, Zhang W, Han Y, Zhao W (2020). Targeting autophagy by natural product ursolic acid for prevention and treatment of osteoporosis. Toxicol Appl Pharm.

[56] Jin X-Y, Chen H, Li D-D, Li A-L, Wang W-Y, Gu W (2019). Design, synthesis, and anticancer evaluation of novel quinoline derivatives of ursolic acid with hydrazide, oxadiazole, and thiadiazole moieties as potent MEK inhibitors. J Enzyme Inhib Med Chem.

[57] Liu P, Du R, Yu X (2019). Ursolic acid exhibits potent anticancer effects in human metastatic melanoma cancer cells (SK-MEL-24) via apoptosis induction, inhibition of cell migration and invasion, cell cycle arrest, and inhibition of mitogen-activated protein kinase (MAPK)/ERK signaling pathway. Med Sci Monit Int Med J Exp Clin Res.

[58] Wu C-C, Cheng C-H, Lee Y-H, Chang I-L, Chen H-Y, Hsieh C-P, Chueh P-J (2016). Ursolic acid triggers apoptosis in human osteosarcoma cells via caspase activation and the ERK1/2 MAPK pathway. J Agric Food Chem.

[59] Guo W, Xu B, Wang X, Zheng B, Du J, Liu S (2020). The analysis of the anti-tumor mechanism of ursolic acid using connectively map approach in breast cancer cells line MCF-7. Cancer Manag Res.

[60] Zhao J, Leng P, Xu W, Sun J-L, Ni B-B, Liu G-W (2021). Investigating the multitarget pharmacological mechanism of ursolic acid acting on colon cancer: a network pharmacology approach. Evid Based Complement Alternat Med.

[61] Zhang X, Li T, Gong ES, Liu RH (2020). Antiproliferative activity of ursolic acid in MDA-MB-231 human breast cancer cells through Nrf2 pathway regulation. J Agric Food Chem.

[62] Cao M, Xiao D, Ding X (2020). The anti-tumor effect of ursolic acid on papillary thyroid carcinoma via suppressing Fibronectin-1. Biosci Biotechnol Biochem.

[63] Li J, Dai C, Shen L (2019). Ursolic acid inhibits epithelial-mesenchymal transition through the Axl/NF-B pathway in gastric cancer cells. Evid Based Complement Alternat Med.

[64] Aguiriano-Moser V, Svejda B, Li ZX, Sturm S, Stuppner H, Ingolic E, Höger H, Siegl V, Meier-Allard N, Sadjak A (2015). Ursolic acid from *Trailliaedoxa gracilis *induces apoptosis in medullary thyroid carcinoma cells. Mol Med Rep.

[65] Peng M, Qiang L, Xu Y, Li C, Li T, Wang J (2018). Modification of cysteine 179 in IKKβ by ursolic acid inhibits titanium-wear-particle-induced inflammation, osteoclastogenesis, and hydroxylapatite resorption. Mol Pharm.

[66] Iqbal J, Abbasi BA, Ahmad R, Mahmood T, Kanwal S, Ali B, Khalil AT, Shah SA, Alam MM, Badshah H (2018). Ursolic acid a promising candidate in the therapeutics of breast cancer: current status and future implications. Biomed Pharmcother.

[67] Mioc M, Milan A, Malița D, Mioc A, Prodea A, Racoviceanu R, Ghiulai R, Cristea A, Căruntu F, Șoica C (2022). Recent advances regarding the molecular mechanisms of triterpenic acids: a review (part I). Int J Mol Sci.

[68] Bose S, Banerjee S, Mondal A, Chakraborty U, Pumarol J, Croley CR, Bishayee A (2020). Targeting the JAK/STAT signaling pathway using phytocompounds for cancer prevention and therapy. Cells.

[69] Kim K, Shin EA, Jung JH, Park JE, Kim DS, Shim BS, Kim S-H (2018). Ursolic acid induces apoptosis in colorectal cancer cells partially via upregulation of microRNA-4500 and inhibition of JAK2/STAT3 phosphorylation. Int J Mol Sci.

[70] Yang M, Hu C, Cao Y, Liang W, Yang X, Xiao T (2021). Ursolic acid regulates cell cycle and proliferation in colon adenocarcinoma by suppressing cyclin B1. Front Pharmacol.

[71] Kang DY, Sp N, Lee J-M, Jang K-J (2021). Antitumor effects of ursolic acid through mediating the inhibition of STAT3/PD-L1 signaling in non-small cell lung cancer cells. Biomedicines.

[72] Liu T, Ma H, Shi W, Duan J, Wang Y, Zhang C, Li C, Lin J, Li S, Lv J (2017). Inhibition of STAT3 signaling pathway by ursolic acid suppresses growth of hepatocellular carcinoma. Int J Oncol.

[73] Kang DY, Sp N, Jang K-J, Jo ES, Bae SW, Yang YM (2022). Antitumor effects of natural bioactive ursolic acid in embryonic cancer stem cells. J Oncol.

[74] Lin W, Ye H (2020). Anticancer activity of ursolic acid on human ovarian cancer cells via ROS and MMP mediated apoptosis, cell cycle arrest and downregulation of PI3K/AKT pathway. J BUON.

[75] Lee N-R, Meng RY, Rah S-Y, Jin H, Ray N, Kim S-H, Park BH, Kim SM (2020). Reactive oxygen species-mediated autophagy by ursolic acid inhibits growth and metastasis of esophageal cancer cells. Int J Mol Sci.

[76] Wang M, Yu H, Wu R, Chen ZY, Hu Q, Zhang YF, Gao SH, Zhou GB (2020). Autophagy inhibition enhances the inhibitory effects of ursolic acid on lung cancer cells. Int J Mol Med.

[77] Kim GD (2021). Ursolic acid decreases the proliferation of MCF-7 cell-derived breast cancer stem-like cells by modulating the ERK and PI3K/AKT signaling pathways. Prev Nutr Food Sci.

[78] Pham JV, Yilma MA, Feliz A, Majid MT, Maffetone N, Walker JR, Kim E, Cho HJ, Reynolds JM, Song MC (2019). A review of the microbial production of bioactive natural products and biologics. Front Microbiol.

[79] Zhang P, Wang Z, Xu L, Yin X, Hu W (2022). CHEN C: Synergistic antibacterial effect of ursolic acid combined with fusidic acid on *Staphylococcus aureus*. Chin J Clin Pharm Ther.

[80] Martelli G, Giacomini D (2018). Antibacterial and antioxidant activities for natural and synthetic dual-active compounds. Eur J Med Chem.

[81] Do Nascimento PG, Lemos TL, Bizerra AM, Arriaga ÂM, Ferreira DA, Santiago GM, Braz-Filho R, Costa JGM (2014). Antibacterial and antioxidant activities of ursolic acid and derivatives. Molecules.

[82] Jabeen M, Ahmad S, Shahid K, Sadiq A, Rashid U (2018). Ursolic acid hydrazide based organometallic complexes: synthesis, characterization, antibacterial, antioxidant, and docking studies. Front Chem.

[83] Zhao W-W, Zan K, Wu J-Y, Gao W, Yang J, Ba Y-Y, Wu X, Chen X-Q (2019). Antibacterial triterpenoids from the leaves of *Ilex hainanensis*. Nat Prod Res.

[84] Sycz Z, Tichaczek-Goska D, Wojnicz D (2022). Anti-planktonic and anti-biofilm properties of pentacyclic triterpenes—asiatic acid and ursolic acid as promising antibacterial future pharmaceuticals. Biomolecules.

[85] Sifaoui I, Rodríguez-Expósito RL, Reyes-Batlle M, Rizo-Liendo A, Piñero JE, Bazzocchi IL, Lorenzo-Morales J, Jiménez IA (2019). Ursolic acid derivatives as potential agents against Acanthamoeba spp. Pathogens.

[86] Al-Kuraishy HM, Al-Gareeb AI (2022). Batiha GE-S: the possible role of ursolic acid in Covid-19: a real game changer. Clin Nutr ESPEN.

[87] Al-Kuraishy HM, Al-Gareeb AI, Negm WA, Alexiou A, Batiha GE-S (2022). Ursolic acid and SARS-CoV-2 infection: a new horizon and perspective. Inflammopharmacology.

[88] Gao Y, Shang Q, Li W, Guo W, Stojadinovic A, Mannion C, Man Y-g, Chen T (2020). Antibiotics for cancer treatment: a double-edged sword. J Cancer.

[89] Hernández DL (2016). Use of antibiotics, gut microbiota, and risk of type 2 diabetes: epigenetics regulation. J Clin Endocrinol Metab.

[90] Sommer F, Anderson JM, Bharti R, Raes J, Rosenstiel P (2017). The resilience of the intestinal microbiota influences health and disease. Nat Rev Microbiol.

[91] Biswas T, Dwivedi UN (2019). Plant triterpenoid saponins: biosynthesis, in vitro production, and pharmacological relevance. Protoplasma.

[92] Simpson DS, Oliver PL (2020). ROS generation in microglia: understanding oxidative stress and inflammation in neurodegenerative disease. Antioxidants.

[93] Gudoityte E, Arandarcikaite O, Mazeikiene I, Bendokas V, Liobikas J (2021). Ursolic and oleanolic acids: plant metabolites with neuroprotective potential. Int J Mol Sci.

[94] Rai SN, Zahra W, Singh SS, Birla H, Keswani C, Dilnashin H, Rathore AS, Singh R, Singh RK, Singh SP (2019). Anti-inflammatory activity of ursolic acid in MPTP-induced parkinsonian mouse model. Neurotox Res.

[95] Habtemariam S (2019). Antioxidant and anti-inflammatory mechanisms of neuroprotection by ursolic acid: addressing brain injury, cerebral ischemia, cognition deficit, anxiety, and depression. Oxid Med Cell Longev.

[96] Naß J, Abdelfatah S, Efferth T (2021). The triterpenoid ursolic acid ameliorates stress in Caenorhabditis elegans by affecting the depression-associated genes skn-1 and prdx2. Phytomedicine.

[97] Li S-J, Liu Q, He X-B, Liu J-P, Liu X-L, Hu J, Tang Z-P, Peng Q-Y, Cui L-J, Zhang H-N (2020). *Pyrola incarnata* demonstrates neuroprotective effects against β-amyloid-induced memory impairment in mice. Bioorg Med Chem Lett.

[98] Wang N, Wang E, Wang R, Muhammad F, Li T, Yue J, Zhou Y, Zhi D, Li H (2022). Ursolic acid ameliorates amyloid β-induced pathological symptoms in Caenorhabditis elegans by activating the proteasome. Neurotoxicology.

[99] Salau VF, Erukainure OL, Ayeni G, Ibeji CU, Islam MS (2021). Modulatory effect of ursolic acid on neurodegenerative activities in oxidative brain injury: An ex vivo study. J Food Biochem.

[100] Alam M, Ali S, Ahmed S, Elasbali AM, Adnan M, Islam A, Hassan MI, Yadav DK (2021). Therapeutic potential of ursolic acid in cancer and diabetic neuropathy diseases. Int J Mol Sci.

[101] Balcazar N, Betancur LI, Muñoz DL, Cabrera FJ, Castaño A, Echeverri LF, Acin S (2021). Ursolic acid lactone obtained from *Eucalyptus tereticornis* increases glucose uptake and reduces inflammatory activity and intracellular neutral fat: an in vitro study. Molecules.

[102] Liang Y, Niu Q, Zhao Y (2021). Pharmacological research progress of ursolic acid for the treatment of liver diseases. Tradit Med Res.

[103] Seo DY, Lee SR, Heo J-W, No M-H, Rhee BD, Ko KS, Kwak H-B, Han J (2018). Ursolic acid in health and disease. Korean J Physiol Pharmacol.

[104] Oboh M, Govender L, Siwela M, Mkhwanazi BN (2021). Anti-diabetic potential of plant-based pentacyclic triterpene derivatives: progress made to improve efficacy and bioavailability. Molecules.

[105] Kim G-H, Kan S-Y, Kang H, Lee S, Ko HM, Kim JH, Lim J-H (2019). Ursolic acid suppresses cholesterol biosynthesis and exerts anti-cancer effects in hepatocellular carcinoma cells. Int J Mol Sci.

[106] Xu H-l, Wang X-t, Cheng Y, Zhao J-g, Zhou Y-j, Yang J-j, Qi M-y (2018). Ursolic acid improves diabetic nephropathy via suppression of oxidative stress and inflammation in streptozotocin-induced rats. Biomed Pharmacother.

[107] Mlala S, Oyedeji AO, Gondwe M, Oyedeji OO (2019). Ursolic acid and its derivatives as bioactive agents. Molecules.

[108] Alqahtani AS, Ullah R, Shahat AA (2022). Bioactive constituents and toxicological evaluation of selected antidiabetic medicinal plants of Saudi Arabia. Evid Based Complement Alternat Med.

[109] EmF Khusnutdinova, Smirnova IE, GnV Giniyatullina, NyI Medvedeva, Yamansarov EY, Kazakov DV, Kazakova OB, Linh PT, Viet DQ, Huong DT (2016). Inhibition of alpha-glucosidase by synthetic derivatives of lupane, oleanane, ursane and dammarane triterpenoids. Nat Prod Commun.

[110] Khusnutdinova EF, Smirnova IE, Kazakova OB, Petrova AV, Ha NTT, Viet DQ (2017). Synthesis and evaluation of 2, 3-indolotriterpenoids as new α-glucosidase inhibitors. Med Chem Res.

[111] Tang S, Fang C, Liu Y, Tang L, Xu Y (2022). Anti-obesity and anti-diabetic effect of ursolic acid against streptozotocin/high fat induced obese in diabetic rats. J Oleo Sci.

[112] Vadivelan R, Krishnan RG, Kannan R (2019). Antidiabetic potential of asparagus racemosus Willd leaf extracts through inhibition of α-amylase and α-glucosidase. J Tradit Complement Med.

[113] Bacanlı M, Aydın S, Anlar HG, Çal T, Arı N, Bucurgat ÜÜ, Başaran AA, Başaran N (2018). Can ursolic acid be beneficial against diabetes in rats?. Turk J Biochem.

[114] Wang X-t, Gong Y, Zhou B, Yang J-j, Cheng Y, Zhao J-g, Qi M-y (2018). Ursolic acid ameliorates oxidative stress, inflammation and fibrosis in diabetic cardiomyopathy rats. Biomed Pharmacothe.

[115] Bacanlı M. 2020 Limonene and ursolic acid in the treatment of diabetes: Citrus phenolic limonene, triterpenoid ursolic acid, antioxidants and diabetes. In: Diabetes. Elsevier. Amsterdam 275–283.

[116] Adebayo S, Amoo S (2019). South African botanical resources: a gold mine of natural pro-inflammatory enzyme inhibitors?. S Afr J Bot.

[117] Luan M, Wang H, Wang J, Zhang X, Zhao F, Liu Z, Meng Q (2022). Advances in Anti-inflammatory Activity, Mechanism and Therapeutic Application of Ursolic Acid. Mini Rev Med Chem.

[118] Zhou J-X, Wink M (2019). Evidence for anti-inflammatory activity of isoliquiritigenin, 18β glycyrrhetinic acid, ursolic acid, and the traditional Chinese medicine plants Glycyrrhiza glabra and eriobotrya japonica, at the molecular level. Medicines.

[119] Zhao J, Zheng H, Sui Z, Jing F, Quan X, Zhao W, Liu G (2019). Ursolic acid exhibits anti-inflammatory effects through blocking TLR4-MyD88 pathway mediated by autophagy. Cytokine.

[120] Zhang T-Y, Li C-S, Li P, Bai X-Q, Guo S-Y, Jin Y, Piao S-J. 2020 Synthesis and evaluation of ursolic acid-based 1, 2, 4-triazolo [1, 5-a] pyrimidines derivatives as anti-inflammatory agents. Mol Divers. 1–12 10.1007/s11030-020-10154-7 33200293

[121] Zhang T-Y, Li C-S, Cao L-T, Bai X-Q, Zhao D-H, Sun S-M (2022). New ursolic acid derivatives bearing 1, 2, 3-triazole moieties: design, synthesis and anti-inflammatory activity in vitro and in vivo. Mol Divers.

[122] Ahmad A, Abuzinadah MF, Alkreathy HM, Banaganapalli B, Mujeeb M (2018). Ursolic acid rich ocimum sanctum L leaf extract loaded nanostructured lipid carriers ameliorate adjuvant induced arthritis in rats by inhibition of COX-1, COX-2, TNF-α and IL-1: pharmacological and docking studies. PLoS ONE.

[123] Wang C, Gao Y, Zhang Z, Chen C, Chi Q, Xu K, Yang L (2020). Ursolic acid protects chondrocytes, exhibits anti-inflammatory properties via regulation of the NF-κB/NLRP3 inflammasome pathway and ameliorates osteoarthritis. Biomed Pharmacother.

[124] Zhao C, Yin S, Dong Y, Guo X, Fan L, Ye M, Hu H (2013). Autophagy-dependent EIF2AK3 activation compromises ursolic acid-induced apoptosis through upregulation of MCL1 in MCF-7 human breast cancer cells. Autophagy.

[125] Yeh CT, Wu CH, Yen GC (2010). Ursolic acid, a naturally occurring triterpenoid, suppresses migration and invasion of human breast cancer cells by modulating c-Jun N-terminal kinase, Akt and mammalian target of rapamycin signaling. Mol Nutr Food Res.

[126] Singh DD, Verma R, Tripathi SK, Sahu R, Trivedi P, Yadav DK (2021). Breast cancer transcriptional regulatory network reprogramming by using the CRISPR/Cas9 system: an oncogenetics perspective. Curr Top Med Chem.

[127] De Angel RE, Smith SM, Glickman RD, Perkins SN, Hursting SD (2010). Antitumor effects of ursolic acid in a mouse model of postmenopausal breast cancer. Nutr Cancer.

[128] Jin H, Pi J, Yang F, Jiang J, Wang X, Bai H, Shao M, Huang L, Zhu H, Yang P (2016). Folate-chitosan nanoparticles loaded with ursolic acid confer anti-breast cancer activities in vitro and in vivo. Sci Rep.

[129] Lin L, Liu A, Peng Z, Lin H-J, Li P-K, Li C, Lin J (2011). STAT3 is necessary for proliferation and survival in colon cancer-initiating cellsstat3 in colorectal cancer-initiating cells. Can Res.

[130] Wang W, Zhao C, Jou D, Lü J, Zhang C, Lin L, Lin J (2013). Ursolic acid inhibits the growth of colon cancer-initiating cells by targeting STAT3. Anticancer Res.

[131] Hutcheson IR, Knowlden JM, Madden T-A, Barrow D, Gee JM, Wakeling AE, Nicholson RI (2003). Oestrogen receptor-mediated modulation of the EGFR/MAPK pathway in tamoxifen-resistant MCF-7 cells. Breast Cancer Res Treat.

[132] Yu FY, Tu Y, Deng Y, Guo C, Ning J, Zhu Y, Lv X, Ye H (2016). MiR-4500 is epigenetically downregulated in colorectal cancer and functions as a novel tumor suppressor by regulating HMGA2. Cancer Biol Ther.

[133] Richerioux N, Blondeau C, Wiedemann A, Rémy S, Vautherot J-F, Denesvre C (2012). Rho-ROCK and Rac-PAK signaling pathways have opposing effects on the cell-to-cell spread of marek’s disease virus. PLoS ONE.

[134] Mu D, Zhou G, Li J, Su B, Guo H (2018). Ursolic acid activates the apoptosis of prostate cancer via ROCK/PTEN mediated mitochondrial translocation of cofilin-1. Oncol Lett.

[135] Wu B, Wang X, Chi Z-f, Hu R, Zhang R, Yang W, Liu Z-g (2012). Ursolic acid-induced apoptosis in K562 cells involving upregulation of PTEN gene expression and inactivation of the PI3K/Akt pathway. Arch Pharm Res.

[136] Park J-H, Kwon H-Y, Sohn EJ, Kim KA, Kim B, Jeong S-J, Koo JS, Kim S-H (2013). Inhibition of Wnt/β-catenin signaling mediates ursolic acid-induced apoptosis in PC-3 prostate cancer cells. Pharmacol Rep.

[137] Li S, Wu R, Wang L, Dina Kuo HC, Sargsyan D, Zheng X, Wang Y, Su X, Kong AN (2022). Triterpenoid ursolic acid drives metabolic rewiring and epigenetic reprogramming in treatment/prevention of human prostate cancer. Mol Carcinog.

[138] Petermann A, Miene C, Schulz-Raffelt G, Palige K, Hölzer J, Glei M, Böhmer FD (2009). GSTT2, a phase II gene induced by apple polyphenols, protects colon epithelial cells against genotoxic damage. Mol Nutr Food Res.

[139] Wang C, Shu L, Zhang C, Li W, Wu R, Guo Y, Yang Y, Kong AN (2018). Histone methyltransferase Setd7 regulates Nrf2 signaling pathway by phenethyl isothiocyanate and ursolic acid in human prostate cancer cells. Mol Nutr Food Res.

[140] Lin JH, Chen SY, Lu CC, Lin JA, Yen GC (2020). Ursolic acid promotes apoptosis, autophagy, and chemosensitivity in gemcitabine-resistant human pancreatic cancer cells. Phytother Res.

[141] Li Z-Y, Chen S-Y, Weng M-H, Yen G-C (2021). Ursolic acid restores sensitivity to gemcitabine through the RAGE/NF-κB/MDR1 axis in pancreatic cancer cells and in a mouse xenograft model. J Food Drug Anal.

[142] Mullen PJ, Yu R, Longo J, Archer MC, Penn LZ (2016). The interplay between cell signalling and the mevalonate pathway in cancer. Nat Rev Cancer.

[143] Seebaluck R, Gurib-Fakim A, Mahomoodally F (2015). Medicinal plants from the genus *Acalypha* (Euphorbiaceae)–a review of their ethnopharmacology and phytochemistry. J Ethnopharm.

[144] Wen J-h, Wei X-h, Sheng X-y, Zhou D-q, Peng H-w, Lu Y-n, Zhou J (2015). Effect of ursolic acid on breast cancer resistance protein-mediated transport of rosuvastatin in vivo and vitro. Chin Med Sci J.

[145] Kim K-A, Lee J-S, Park H-J, Kim J-W, Kim C-J, Shim I-S, Kim N-J, Han S-M, Lim S (2004). Inhibition of cytochrome P450 activities by oleanolic acid and ursolic acid in human liver microsomes. Life Sci.

[146] Yang J, Hao C, Yang D, Shi D, Song X, Luan X, Hu G, Yan B (2010). Pregnane X receptor is required for interleukin-6-mediated down-regulation of cytochrome P450 3A4 in human hepatocytes. Toxicol Lett.

[147] Jinhua W, Ying Z, Yuhua L (2020). PXR–ABC drug transporters/CYP-mediated ursolic acid transport and metabolism in vitro and vivo. Arch Pharm.

[148] Nguyen HN, Ullevig SL, Short JD, Wang L, Ahn YJ, Asmis R (2021). Ursolic acid and related analogues: triterpenoids with broad health benefits. Antioxidants.

[149] Sun Q, He M, Zhang M, Zeng S, Chen L, Zhou L, Xu H (2020). Ursolic acid: a systematic review of its pharmacology, toxicity and rethink on its pharmacokinetics based on PK-PD model. Fitoterapia.

[150] Liu C-H, Wong SH, Tai C-J, Tai C-J, Pan Y-C, Hsu H-Y, Richardson CD, Lin L-T (2021). Ursolic acid and its nanoparticles are potentiators of oncolytic measles virotherapy against breast cancer cells. Cancers.

[151] Kahnt M, Fischer L, Al-Harrasi A, Csuk R (2018). Ethylenediamine derived carboxamides of betulinic and ursolic acid as potential cytotoxic agents. Molecules.

